# The Role of AIRE in the Immunity Against *Candida Albicans* in a Model of Human Macrophages

**DOI:** 10.3389/fimmu.2018.00567

**Published:** 2018-03-21

**Authors:** Jose Antonio Tavares de Albuquerque, Pinaki Prosad Banerjee, Angela Castoldi, Royce Ma, Nuria Bengala Zurro, Leandro Hideki Ynoue, Christina Arslanian, Marina Uchoa Wall Barbosa-Carvalho, Joya Emilie de Menezes Correia-Deur, Fernanda Guimarães Weiler, Magnus Regios Dias-da-Silva, Marise Lazaretti-Castro, Luis Alberto Pedroza, Niels Olsen Saraiva Câmara, Emily Mace, Jordan Scott Orange, Antonio Condino-Neto

**Affiliations:** ^1^Department of Immunology, Institute of Biomedical Sciences, University of São Paulo, São Paulo, Brazil; ^2^Center for Human Immunobiology, Texas Children’s Hospital, Houston, TX, United States; ^3^Department of Pediatrics, Baylor College of Medicine, Houston, TX, United States; ^4^Escola Paulista de Medicina, Universidade Federal de São Paulo, São Paulo, Brazil; ^5^Colegio de Ciencias de la Salud, Escuela de Medicina, Hospital de los Valles, Universidad San Francisco de Quito, Quito, Ecuador; ^6^Institute of Tropical Medicine, University of São Paulo, São Paulo, Brazil

**Keywords:** APECED, AIRE, *C. albicans*, hyphae, macrophages, receptor recruitment, Dectin receptor

## Abstract

Autoimmune-polyendocrinopathy-candidiasis-ectodermal dystrophy (APECED) is a primary immunodeficiency caused by mutations in the autoimmune regulator gene (*AIRE*). Patients with AIRE mutations are susceptible to *Candida albicans* infection and present with autoimmune disorders. We previously demonstrated that cytoplasmic AIRE regulates the Syk-dependent Dectin-1 pathway. In this study, we further evaluated direct contact with fungal elements, synapse formation, and the response of macrophage-like THP-1 cells to *C. albicans* hyphae to determine the role of AIRE upon Dectin receptors function and signaling. We examined the fungal synapse (FS) formation in wild-type and AIRE-knockdown THP-1 cells differentiated to macrophages, as well as monocyte-derived macrophages from APECED patients. We evaluated Dectin-2 receptor signaling, phagocytosis, and cytokine secretion upon hyphal stimulation. AIRE co-localized with Dectin-2 and Syk at the FS upon hyphal stimulation of macrophage-like THP-1 cells. AIRE-knockdown macrophage-like THP-1 cells exhibited less Dectin-1 and Dectin-2 receptors accumulation, decreased signaling pathway activity at the FS, lower *C. albicans* phagocytosis, and less lysosome formation. Furthermore, IL-1β, IL-6, or TNF-α secretion by AIRE-knockdown macrophage-like THP-1 cells and AIRE-deficient patient macrophages was decreased compared to control cells. Our results suggest that AIRE modulates the FS formation and hyphal recognition and help to orchestrate an effective immune response against *C. albicans*.

## Introduction

Immunocompromised patients affected by fungal infections experience high morbidity and mortality. Most of these infections are caused by opportunistic yeasts, such as *Candida spp*., present in the skin and mucous membranes (1). It is well established that the recognition and destruction of *Candida* by phagocytic cells are pivotal (2). The interactions between different receptors on phagocytic cells and components of the fungal cell wall are known to trigger phagocytosis and subsequent immune responses (3, 4). However, the receptors directly mediating the initial stage of recognition have not been clearly defined.

Although many immunodeficient patients are susceptible to fungal infections, some deficiencies have a signature disease associated with yeast (5–8). Patients with CARD9 deficiency are susceptible to invasive fungal diseases and chronic mucocutaneous candidiasis (CMC) as a result of primary immune dysfunction (9, 10). Patients with inborn errors of IL-17F, IL-17RA, or at the adaptor protein from IL-17 receptor, ACT1, which abolish the TH17 response, present clinically with CMC (11, 12). Gain-of-function (GOF) mutations in signal transducer and activator of transcription 1 (STAT1) impair dephosphorylation of STAT1 in response to IFN-γ, IFN-α/β, and IL-27 stimulation. This leads to defects of IL-17-producing T cells, and many of these patients develop severe CMC (5, 13). STAT3 plays a central role in signal transduction downstream of IL-6, IL-10, IL-17, IL-22, IL-23, and IL-27 cytokines. STAT3-deficient patients have severely decreased frequency of circulating IL-17A- and IL-22-producing T cells, and as a result also develop CMC (5, 14). The Y238X polymorphism in Dectin-1 do not result in immune deficiencies but it increases predisposition to fungal infections due to low production of cytokines by innate immune cells and a functional defect in T helper (TH) 17 responses (15, 16). Finally, patients with autoimmune polyendocrinopathy-candidiasis-ectodermal dystrophy (APECED) may present with high titers of neutralizing autoantibodies against IL-17A, IL-17F, and IL-22 and subsequent CMC (17, 18).

Autoimmune polyendocrinopathy-candidiasis-ectodermal dystrophy is a rare syndrome with a worldwide incidence of 1:100,000, albeit higher incidence in certain ethnic groups such as Italians, Iranian Jews, and Finns (1:9,000, 1:14,000, and 1:25,000, respectively) (17, 19). APECED is caused by mutations in the autoimmune regulator (*AIRE*) gene, which encodes a transcription factor involved in thymic antigen expression and T cell education (19–21). However, many studies have shown that AIRE is expressed in extra-thymic cells. These include peripheral lymphoid tissues and monocytes and dendritic cells, and there is evidence that AIRE can modulate the inflammatory response (22–24).

Interestingly, CMC, typically affecting the oral, vaginal, and esophageal mucosa, is the sole infectious disease seen in AIRE-deficient patients (17, 18, 21, 25). This susceptibility can be potentially attributed to the production of autoantibodies against IL-17 and IL-22 cytokines in APECED patients (17, 21). However, some AIRE-deficient patients do not produce autoantibodies against IL-17A, IL17F, and IL-22 but still have CMC, similarly the presence of cytokine autoantibodies does not correlate with the presence of CMC (18, 26). This suggests that other factors may be involved in the profound susceptibility to develop CMC in APECED patients.

Dectin-1 and Dectin-2 receptors recognize yeast and hyphal forms of *C. albicans*, respectively (27). The balance in the activation of these receptors stimulates the phagocytic activity, lysosomal activation, and production of cytokines, which in turn contribute to the anti-fungal response including the generation of TH1 and TH17 cells (28). Although some studies have shown that susceptibility of APECED patients to candidiasis is related to defects in innate immune cells, the molecular mechanisms potentially impairing mononuclear phagocytes remain unclear.

We have previously demonstrated that cytoplasmic AIRE regulates the Syk-dependent Dectin-1 pathway and TNF-α secretion by monocytes in APECED patients (23). The Syk-dependent pathway, however, is shared by other receptors involved in defense against fungal infections, such as Dectin-2 (28). *C. albicans* hyphae possess low concentrations of β-glucan and high concentrations of α-mannan exposed in the cell wall that are recognized, respectively, by Dectin-1 and Dectin-2 (29, 30). Thus, the recruitment and activation of Dectin-1 and Dectin-2 to the interface formed with fungal elements, which we term as fungal synapse (FS), appears to be crucial for an effective response against *C. albicans*.

We hypothesized that AIRE is important for an effective response against *C. albicans* and that this response involves Dectin-1 and Dectin-2 receptors at the FS, influencing further steps of the immunological response.

## Materials and Methods

### Patients and Healthy Donors

We selected adult APECED patients presenting with CMC and autoimmunity, as confirmed by genetic analysis of the *AIRE* gene; two of these patients have a Pro326Leu substitution, one has two deleted residues (exon 5 Ser187 and exon 9 Gln358) and another stop codon in exon 5. Blood samples were collected from patients and healthy donors and were then processed and shipped according to the protocols approved by the Institutional Ethics Committee, the Ministry of Health of Brazil, and the Helsinki Convention.

### Reagents

The following reagents were used: puromycin, piceatannol, and polybrene (Sigma-Aldrich); RPMI 1640 culture medium and Dulbecco’s PBS (Invitrogen); anti-Dectin-1, anti-Dectin-2 (H-46), anti-AIRE (H-300 and C-2), anti-Syk (4D10), anti-CARD9 (C-17) (Santa Cruz Biotechnology); anti-Dectin-1 (AF1756), anti-Dectin-2 (AF3114) (R&D Systems); and human IgG (Abcam 90285). Lentiviral particles specific for AIRE knockdown and control shRNA Lentiviral particles (shRNA; sc-37669-V and sc-108080) (Santa Cruz Biotechnology) were used in the transduction of THP-1 cell.

### Culture Conditions

The myelomonocytic THP-1 cell line was cultured in RPMI 1640 supplemented with 10% inactivated fetal bovine serum (iFBS), 2 mM l-glutamine, 10 U/mL penicillin, 100 µg/mL streptomycin, 10 mM HEPES, 2 mM sodium pyruvate, and 100 µM MEM non-essential amino acids, in a humid 5% CO_2_ atmosphere at 37°C. 2.5 × 10^6^ THP-1 cells were differentiated to macrophage-like THP-1 cells using 100 U/mL IFN-γ and 1000 U/mL TNF-α for 24–48 h in 6-well plates, following overnight resting in RPMI media. This time point was selected based upon detection of AIRE protein by western blotting (data not shown). Only adherent cells were used in the experiments. Peripheral blood mononuclear cells (PBMCs) from healthy donors and AIRE patients were purified using a Ficoll Paque gradient (GE Healthcare Life Sciences). Human monocytes were isolated by adherence and differentiated into macrophages by incubation with 5 ng/mL GM-CSF (R&D Systems) for 6 days. The *C. albicans* strain used was ATCC SC5314 (kindly provided by Dr. Joachim Morschhauser from Institut Fur Molekulare Infektionsbiologie, Wurzburg, Germany) (31), and it was cultured in Sabouraud medium with 10% iFBS for 3 h to induce hyphal differentiation.

### Transduction of THP-1 Cells With Lentiviral Particles and AIRE Overexpression in HEK293T Cells

2 × 10^4^ THP-1 cells were cultured in supplemented RPMI medium in 96-well plates for 24 h. Then, 8 mg/mL polybrene was added prior to cell transduction with lentiviral particles at a multiplicity of infection (MOI) of 1:10. Cells were incubated for 5 h, and then the culture medium was replaced with fresh medium. After 48 h culture stabilization, fresh medium containing 1.5 mg/mL puromycin was added for 72 h to select transduced cells. The puromycin concentration was then increased to 3 mg/mL, and the cells were incubated for an additional 72 h. AIRE expression in the transduced clones was monitored by western blotting, flow cytometry, and RT-PCR, using anti-AIRE (H-300 and C-2), donkey anti-rabbit Alexa Fluor 647 (a31573) (Molecular Probes), and human AIRE primers (sc-37669-PR). For overexpression of AIRE, 3 × 10^6^ HEK293T cells were transfected with pLenti-AIRE (NM_000383) Human Tagged ORF, mGFP tagged (RC213497L2, OriGene) using FuGENE 6 Transfection Reagent (Promega) for overexpression of AIRE.

### Complementary DNA Preparation and Real-Time PCR

3 × 10^6^ macrophages-like THP-1 cells stimulated with hyphae for 10, 20, or 30 min were submitted to RNA extraction using Trizol reagent (Life Technologies). Preparation of complementary DNA (cDNA) was performed using 2 μg of RNA, 0.8 µL of reverse transcriptase M-MLV, 4 μL of 5× Reaction Buffer M-MLV, 2 μL of 10 mM dNTPs, 32 U/μL of RiboLock™ Ribonuclease Inhibitor (Fermentas), and 320 ng of oligo-dT primer (Integrated DNA Technologies). This mixture was incubated at 42°C for 60 min and then at 70°C for 10 min. cDNA was stored at −20°C. After preparation of cDNA, quantification of gene expression by real-time PCR was performed. Amplification conditions were standardized for each transcript. A comparative relationship between reaction cycles (CT) was used to determine gene expression relative to *PPIA* control (housekeeping gene). The detection of the gene of interest was performed using Quantstudio apparatus (Applied Biosystems). Commercially available probes (Life Technologies) were used for *PPIA* (Hs_99999904). The following sequences of primers, detected using SybrGreen^®^ (Life Technologies) were also used for this study: KiCqStart Primers Human H_*Clec7a* (*Dectin*-1) (NM_022570), H_*Syk* (NM_001135052), H-*Card9* (NM_052813), H_*Clec6* (*Dectin*-2) (NM_001007033), and H_*AIRE* (NM_000383) (Sigma Aldrich). The quantification method was 2 − ΔΔCt (32) using unstimulated THP-1 samples as a normalizer.

### Western Blotting and Immunoprecipitation

Wild-type and AIRE-knockdown macrophage-like THP-1 cells (3 × 10^6^ cells) were stimulated with Candida hyphae. Lysates were obtained using Pierce RIPA buffer. The protein concentrations were determined using a Pierce BCA Protein Assay Kit (Life Technologies), and equivalent amounts of protein were subjected to SDS-PAGE. The membrane was incubated with primary antibody for 1 h, followed by incubation with an IRDye 800CW, IRDye 680RD (LI-COR), or horseradish peroxidase (Sigma Aldrich) secondary antibody. Bands were visualized with an Odyssey^®^ CLx Infrared Imaging System (LI-COR) or ImageQuant LAS 500 (GE Healthcare Life Sciences). 18 × 10^6^ macrophage-like THP-1 cells, control, or stimulated with hyphae was lysed with immunoprecipitation buffer (500 mM Tris pH7.4; 500 mM NaCl; 500 mM EDTA pH8.0; 200 mM EGTA; 1% TRITON X-100). Total protein was quantified using Bradford reagent (BioRad) before immunoprecipitation with anti-AIRE (C-2), anti-Dectin-2 (H-46) (Santa Cruz Biotechnology), and control IgG. Macrophage-like THP-1 cells (2.5 × 10^6^ cells) were stimulated with Candida hyphae for 10, 20, or 30 min. The cytoplasmic fraction was separated using Buffer 1 (10 mM HEPES pH7.9, 50 mM NaCl, 0.5 M sucrose, 0.1 mM EDTA, and 0.5% TRITON X-100). The nuclear pellet was washed using 1 mL Wash Buffer (10 mM HEPES, 60 mM KCl, 1 mM EDTA, 1 mM DTT, 1 mM PMSF), and nuclear fraction extraction was performed using Buffer 2 (20 mM Tris-HCl, 1.5 mM MgCl_2_, 400 mM NaCl, 0,2 mM EDTA, 1 mM PMSF, and 15% Glycerol). Western blotting was performed using anti-AIRE (H-300), anti-AIRE (C-2), anti-CARD9, anti-Syk (4D10), anti-Dectin-1 (Santa Cruz Biotechnology), and anti-Dectin-2 (AF3114) (R&D Systems). Anti-β-Actin (I-19), anti-GAPDH (FL-335) (Santa Cruz Biotechnology), and anti-Lamin B1 (Abcam16048) were used as loading controls.

### Confocal Microscopy

After macrophage differentiation, cells were trypsinized and adjusted to 2 × 10^5^ cells/mL. Cells were then incubated for 45 min at 37°C on poly-l-lysine-treated slides to promote cellular adhesion. Macrophage-like THP-1 cells were subsequently stimulated with *C. albicans* hyphae for different times. Fc receptors were blocked using human IgG to prevent non-specific binding, followed by staining for anti-Dectin-1 (AF1756), anti-Dectin-2 (AF3114) (R&D Systems), or anti-Dectin-2 (H-46) (Santa Cruz Biotechnology). Cells were then fixed with 100 µL CytoFix/CytoPerm (BD Bioscience), according to the manufacturer’s recommendations, and stained for anti-AIRE (H-300), anti-Syk (4D10), or anti-CARD9 (C-17) (Santa Cruz Biotechnology) on ice to reduce non-specific binding. The cells were washed with wash buffer (BD Bioscience) and stained with fluorescence secondary antibody (Molecular Probes). Slides were mounted with Gel Mount (Vectashield) and sealed with nail polish prior to image acquisition using a Zeiss confocal microscopy equipped with Yokogawa CSU-10 spinning disk and Hamamatsu ORCA-ER camera. Confocal micrographs were analyzed by Volocity software (PerkinElmer) to obtain area and mean fluorescence intensity (MFI). 30 cells from 3 independent experiments were measured. All antibodies were validated with a species-specific IgG negative control at the same concentration (Figure S1A in Supplementary Material). To validate the specificity of the AIRE antibody used for confocal microscopy, AIRE-mGFP was overexpressed in HEK293T cells as described earlier. Cells were fixed using CytoFix/CytoPerm (BD Biosciences) or FoxP3/Transcription Factor Fix/Perm solution (Thermo Fisher Scientific) at room temperature and gently washed with Perm wash (BD Biosciences) or Perm buffer (Thermo Fisher Scientific). Staining for GFP was performed with anti-GFP (BioLegend) directly conjugated to AlexaFluor 488 (BioLegend). Staining for AIRE was performed with anti-AIRE (Santa Cruz Biotechnology) followed by goat anti-rabbit IgG conjugated to AlexaFluor 568. Coverslips were mounted using using VectaShield with DAPI (Vector Laboratories). Images were acquired in Leica TCS SP8 laser scanning confocal microscope (Leica Microsystems) using LASAFx software (Figure S1B in Supplementary Material).

### *C. albicans* Phagocytosis and Lysosome Production

Wild-type and AIRE-knockdown macrophage-like THP-1 cells were treated with 10 µg/mL piceatannol (selective Syk inhibitor) or vehicle control and stimulated with hyphae or yeast for 20 or 30 min. To evaluate lysosome production, cells were incubated with LysoTracker Red for 30 min and washed and stimulated with *C. albicans*. Next, the cells were fixed with 4% paraformaldehyde in PBS for 10 min and washed and resuspended in 80 µL PBS. Samples were analyzed by Imagestream X Mark II Imaging Flow Cytometer (Amnis). Flow cytometry image data analysis was performed on LysoTracker/Candida-GFP double positive cells, following by the analysis of the internalization function using IDEAS software (Amnis). The internalization gate was generated using THP-1 macrophages stimulated at 4°C. Human macrophages were stimulated with yeast (MOI 1:5) and hyphae (MOI 1:2) for 30 min on coverslips for phagocytosis evaluations using cells from healthy donors and patients. After being washed, the cells were stained with panoptic dye (Laborclin), and *C. albicans* were counted under a light microscope. As control, human macrophages or macrophage-like THP-1 cells were stimulated with *E. coli* (MOI 1:5) for 30 min, incubated with gentamicin for 45 min, washed and cultured in Luria Bertani agar. The number of colony-forming units (CFU) was counted after overnight incubation at 37°C.

### Cytokine Evaluation

Macrophage-like THP-1 cells were stimulated with hyphae for 6 h. Supernatants were collected and assessed for IL-1β and TNF-α using BioLegend’s LEGENDplex™ kits. Human macrophages were stimulated with hyphae or 100 ng/mL lipopolysaccharide (LPS; Sigma Aldrich), and cytokine secretion was evaluated using IL-6 (eBioscience) and TNF-α (BD Biosciences) ELISA kits. A standard curve was generated using purified protein to obtain a *R*^2^ value of 0.99.

## Results

### FS Formation in Macrophages After Hyphae Stimulation

**Figure 1:**
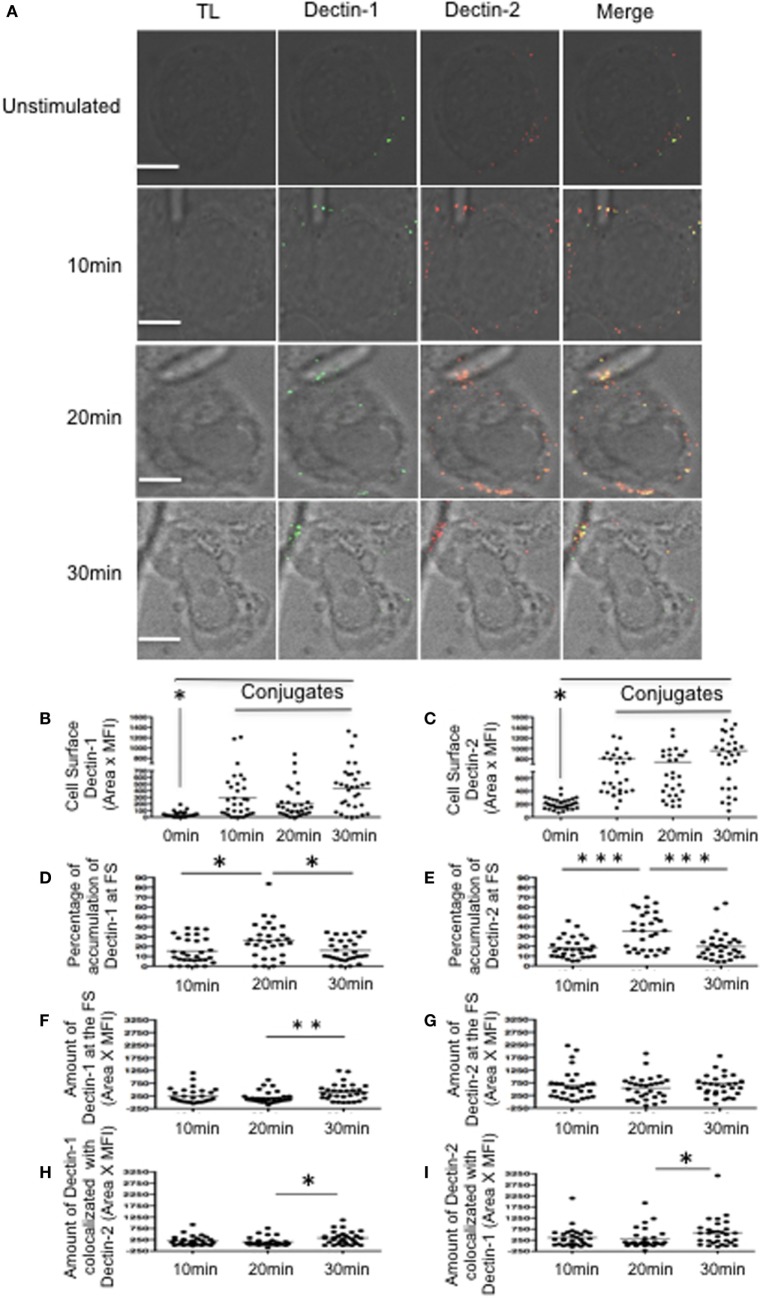
Dynamics of Dectin-1 and Dectin-2 recruitment to the fungal synapse (FS). **(A)** Representative confocal micrographs of macrophage-like THP-1 cells stimulated with *Candida albicans* hyphae for 10, 20, or 30 min to observe the recruitment of Dectin-1 (green) and Dectin-2 (red) receptors to the FS. **(B–I)** Each dot represents a cell used for the measurement indicated. The horizontal bars denote the mean. * *p* < 0.05 as determined by *t*-test. **(B,C)** Amounts of Dectin-1 **(B)** and Dectin-2 **(C)** on the cell surface. **(D,E)** Relative percentages of total Dectin-1 **(D)** and Dectin-2 **(E)** at the FS compared with that in the whole cell. **(F,G)** Amounts of Dectin-1 **(F)** and Dectin-2 **(G)** at the FS. **(H,I)** Synaptic co-localization of Dectin-1 with Dectin 2 **(H)** or of Dectin-2 with Dectin-1 **(I)**. Representative confocal micrographs of 30 cells counted in 3 independent experiments. Scale bar = 5 µm.

To evaluate the FS formation, macrophage-like THP-1 cells were stimulated with *C. albicans* hyphae for 10, 20, or 30 min (Figure 1A). Dectin-1 and Dectin-2 expression increased at the membrane following contact with hyphae compared to unstimulated macrophage-like THP-1 cells (Figures 1B,C). Analysis of macrophage in contact with hyphae demonstrated higher percentages of Dectin-1 and Dectin-2 at the FS after 20 min stimulation compared to levels in the cells as a whole (Figures 1D,E). In addition, expression of Dectin-2 was higher than Dectin-1 at the FS. This was expected since the cell wall of hyphae binds more strongly to Dectin-2 than to Dectin-1. Moreover, the amount of Dectin-1 did increase after 30 min (Figures 1F,G) as did the co-localization of Dectin-1 and Dectin-2 at the FS (Figures 1H,I).

To determine any potential role of Dectin-2 in the recruitment of Dectin-1 to the nascent FS, we first blocked Dectin receptors in macrophage-like THP-1 cells using either an anti-Dectin-1 or anti-Dectin-2 antibody or both for 30 min prior to hyphal stimulation (Figure S2 in Supplementary Material). Blocking Dectin-1 increased the accumulation of Dectin-2 at the FS compared with control IgG-treated macrophages. Since these receptors likely share a Syk-dependent signaling pathway, blocking Dectin-1 may increase signaling promoting Dectin-2 activation. After blocking Dectin-2, however, there was no significant change in Dectin-1 accumulation at the FS (Figures S2A,C in Supplementary Material). To verify that Dectin-1 recruitment was initiated by its own ligation and signaling, we blocked both Dectin receptors before hyphal stimulation. This blockage resulted in decreased accumulation of both receptors at the FS (Figures S2B,D in Supplementary Material). These findings suggest that Dectin-1 recruitment is mainly attributed to its own ligation and activation.

### AIRE Interaction With Dectin-2 and Syk

**Figure 2:**
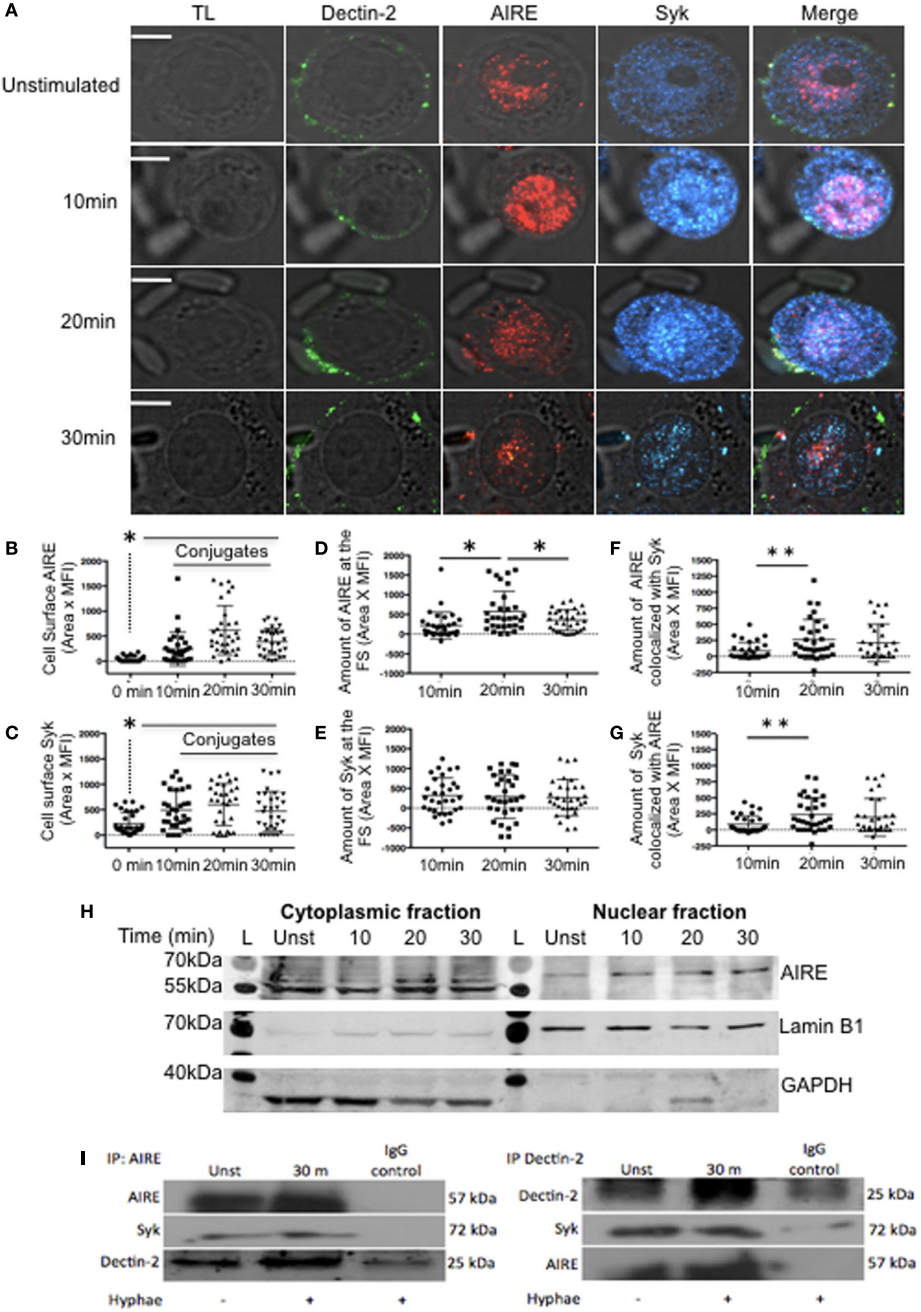
Dectin signaling at fungal synapse (FS). **(A)** Representative confocal micrographs of macrophage-like THP-1 cells, unstimulated (0 min) or stimulated with *C. albicans* hyphae for 10, 20, or 30 min to observe the recruitment of Dectin-2 (green), Syk (blue), and autoimmune regulator (AIRE) (red) to the FS. **(B–G)** Each dot represents a cell used for the measurement indicated. The horizontal bar in each column denotes the mean. *p < 0.05 as determined by the *t*-test. **(B,C)** Amounts of AIRE **(B)** and Syk **(C)** on the cell surface. **(D,E)** Amounts of AIRE **(D)** and Syk **(E)** at the FS (conjugated–unconjugated). **(F,G)** Synaptic co-localization of AIRE with Syk **(F)** or of Syk with AIRE **(G)**. **(H)** Western blotting analysis of AIRE in cytoplasmic and nuclear fractions from macrophage-like THP-1 cells, unstimulated (unst) or stimulated with hyphae for 10, 20, or 30 min. (L) Ladder, GAPDH and Lamin B1 was used as loading control. **(I)** Lysates from macrophage-like THP-1 cells, unst or stimulated with hyphae for 30 min, were immunoprecipitated with AIRE (left panel) or Dectin-2 antibodies (right panel) and with a control antibody (IgG). The immunoprecipitated products were probed for AIRE, Dectin-2, and Syk as indicated. Representative confocal micrographs of 30 cells counted in 3 independent experiments. Scale bar = 5 µm.

First, we investigated whether FS activation is modulating the transcription of *Dectin*-1, *Dectin*-2, *Syk, Card9*, and *AIRE*. We observed that only *Dectin*-2 and *AIRE* mRNA significantly increased the transcription after hyphae stimulation (Figure S3B in Supplementary Material). Since Dectin-2 localizes to the FS and we observed increased mRNA expression levels of *Dectin*-2 and *AIRE*, we next evaluated whether AIRE might be involved in the Dectin-2 signaling pathway. Macrophage-like THP-1 cells were stimulated with hyphae to evaluate AIRE and Syk recruitment to the FS. This stimulation led to considerable co-localization of Dectin-2, Syk, and AIRE (Figure 2A) as well as increased AIRE and Syk expression at the FS (Figures 2B,C). Interestingly, the cell surface expression of AIRE, as well as the accumulation of Dectin-1 and Dectin-2 receptors at the FS, reached peaks at 20 min (Figures 1D,E and 2B). However, the percentage of AIRE accumulated at the FS remained relatively constant across all time points (Figures 2B–D). Thus, AIRE accumulation did not change because its expression was increased throughout the cell and not only at the FS. Although Syk is important for Dectin receptor activation, Syk expression remained consistent at all time points (Figures 2C–E). An increase in AIRE and Syk co-localization, however, was detected after 20 min of hyphal stimulation (Figures 2F,G). Moreover, we separated cytoplasmic and nuclear fractions to confirm AIRE expression in macrophage-like THP-1 cells stimulated with hyphae. AIRE was identified in two isoforms, with higher expression at 20 min in the cytoplasmic fraction while it was increased after hyphae stimulation at the nuclear fraction (Figure 2H). To determine whether Dectin-2, Syk, and AIRE actually physically interact, AIRE or Dectin-2 was immunoprecipitated from hyphae-stimulated macrophage-like THP-1 cells. AIRE was identified in molecular complexes with Dectin-2 and Syk, in macrophage-like THP-1 cells that had been activated with *C. albicans* hyphae (Figure 2I). Moreover, AIRE was increased compared to unstimulated cells. Thus, Dectin-2 localizes to the FS as part of a molecular complex with both Syk and AIRE.

### AIRE Is Required for FS Formation

**Figure 3:**
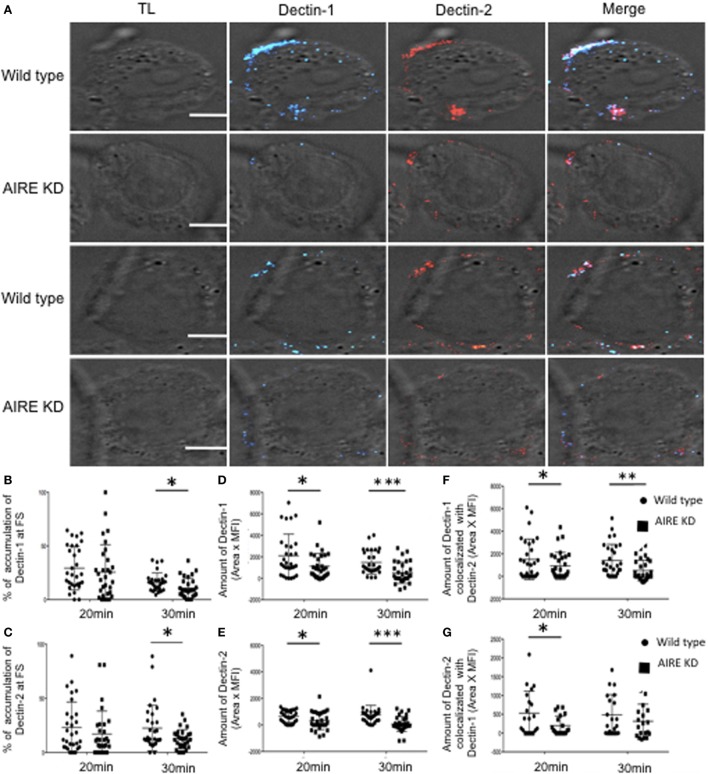
AIRE-mediated recruitment of Dectin receptors to fungal synapse (FS). **(A)** Representative confocal micrographs of wild-type and AIRE-knockdown macrophage-like THP-1 cells stimulated with *C. albicans* hyphae for 20 or 30 min to observe the recruitment of Dectin-1 (blue) and Dectin-2 (red) receptors to the FS. **(B–E)** Each dot represents a cell used for the measurement indicated. The horizontal bars denote the mean. *p < 0.05 as determined by the *t*-test. **(B,C)** Percentages of total Dectin-1 **(B)** and Dectin-2 **(C)** at the FS compared with that in the whole cell. **(D,E)** Amounts of Dectin-1 **(D)** and Dectin-2 **(E)** at the FS (conjugated-unconjugated cells). **(F,G)** Synaptic co-localization of Dectin-1 with Dectin-2 **(F)** or Dectin-2 with Dectin-1 **(G)**. Representative confocal micrographs of 30 cells counted in 3 independent experiments. Scale bar = 5 µm.

**Figure 4:**
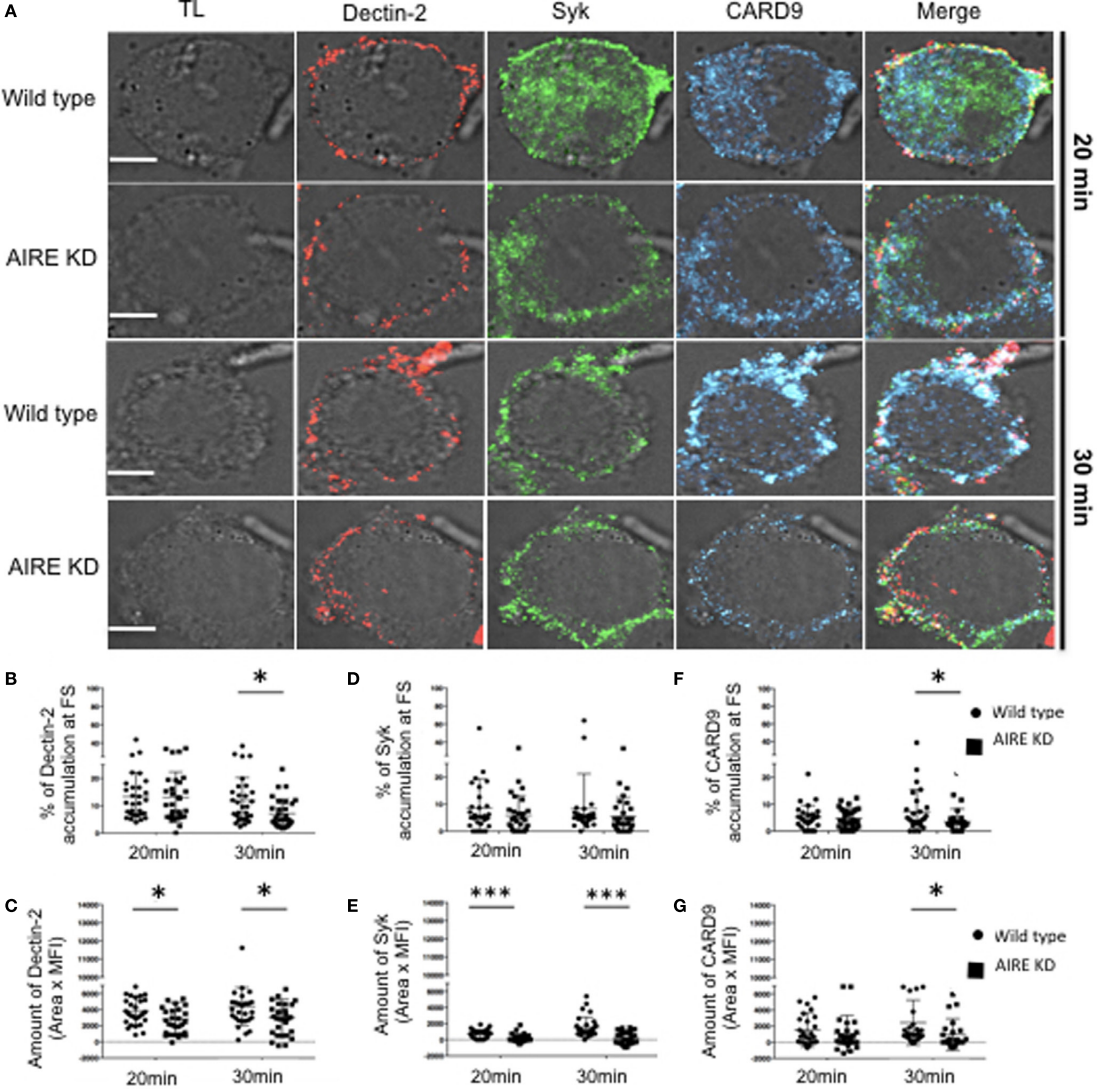
Influence of autoimmune regulator (AIRE) on fungal synapse (FS) formation. **(A)** Representative confocal micrographs of wild-type and AIRE-knockdown macrophage-like THP-1 cells stimulated with *C. albicans* hyphae for 20 or 30 min to observe the recruitment of Dectin-2 (red), Syk (green), and CARD9 (blue) to FS. **(B–E)** Each dot represents a cell used for the measurement indicated. The horizontal bars denote the mean. *p < 0.05 as determined by the *t*-test. **(B,D,F)** Percentages of total Dectin-2 **(B)**, Syk **(D)** and **(F)** CARD9 at the FS compared with those in the whole cell. **(C,E,G)** Amount of Dectin-2 **(C)**, Syk **(E)**, and CARD9 **(G)** at the FS (conjugated–unconjugated cells). Representative confocal micrographs of 30 cells counted in 3 independent experiments. Scale bar = 5 µm.

To evaluate the role of AIRE in macrophages with regard to the FS, AIRE was knocked down in THP-1 cells, and the FS formation compared to that in control cells. There was no overall change in the total expression levels of Dectin-1, Dectin-2, Syk, and CARD9 in the AIRE-knockdown cells (Figures S4A–E in Supplementary Material). Given that these were maintained at physiological levels, we next examined whether AIRE is involved in FS formation. Wild-type and AIRE-knockdown macrophage-like THP-1 cells were stimulated with hyphae for 20 or 30 min, and then Dectin-1 and Dectin-2 recruitment to the FS was analyzed (Figure 3). We observed decreased accumulation of both receptors at the FS in 20 and 30 min in AIRE knockdown macrophages (Figures 3A–G). To evaluate if this has any functional significance, we asked whether protein signaling was affected in AIRE knockdown cells. Importantly, decreased Syk amount was observed at the FS at both time points in AIRE-knockdown macrophages and decreased CARD9 recruitment was identified at 30 min (Figures 4A–G). These results suggest that the delayed FS formation is attributed to decreases in Dectin-1 and Dectin-2 recruitment and protein signaling.

### The Role of AIRE in Macrophage Anti-Fungal Activity

**Figure 5:**
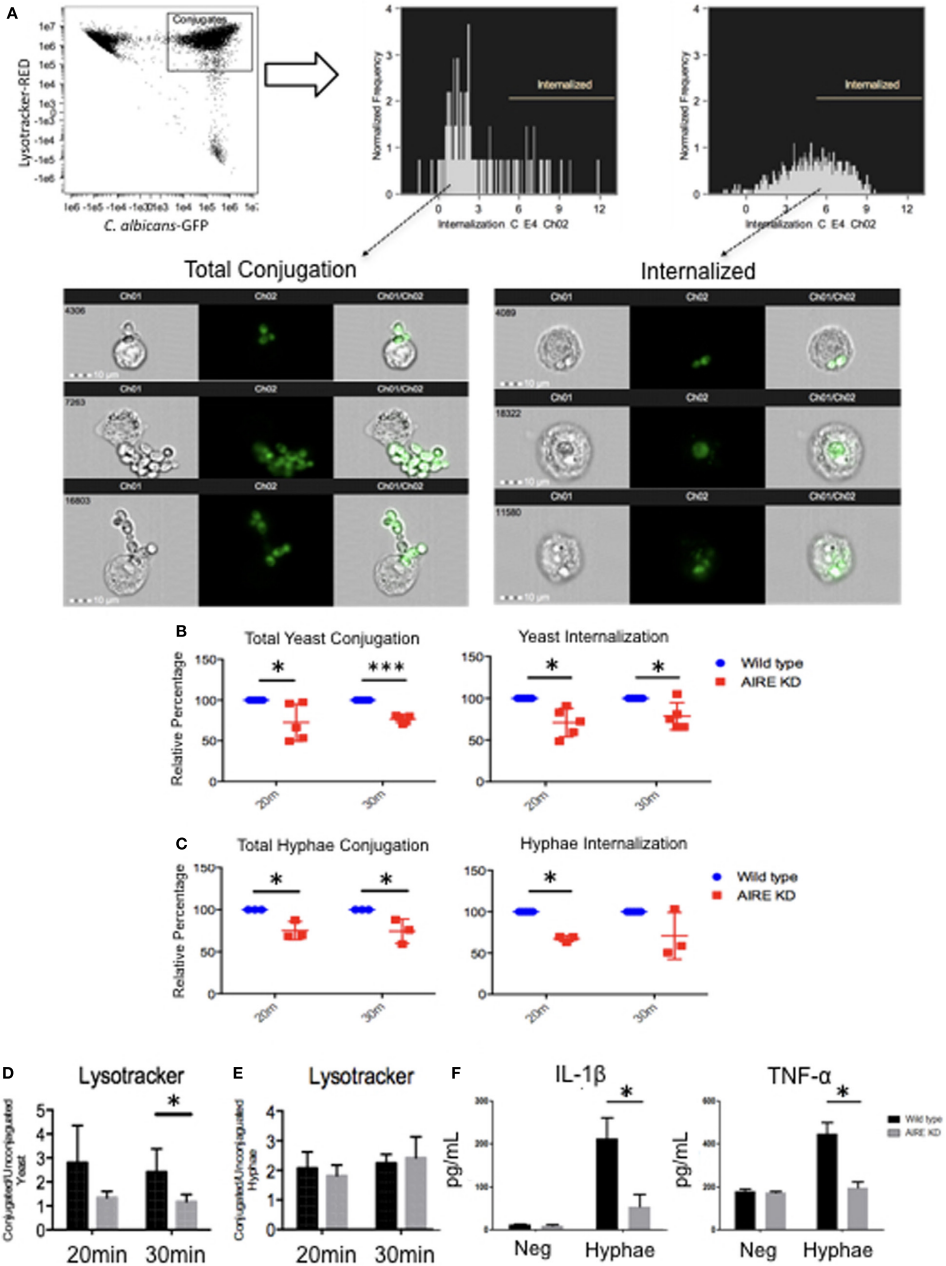
Candidacidal activity in wild-type and AIRE-knockdown macrophage-like THP-1 cells. Wild-type and AIRE-knockdown macrophage-like THP-1 cells were stimulated with yeast [multiplicity of infection (MOI 1:2)] **(B)** or hyphae (MOI 2:1) GFP *C. albicans* **(C)** for 20 or 30 min. **(A)** Image Stream analysis of fungal phagocytosis by macrophages. GFP fluorescence intensities were gated for further analysis. The cells were divided into subpopulations according to the GFP maximum pixels. Internalization gate was performed for cells incubated with yeast or hyphae at 4°C. Each dot represents one independent experiment used for the measurement indicated. Wild-type (blue circle) and AIRE-knockdown macrophage-like THP-1 cells (red square). **(D,E)** Measurement of cytoplasm acidification in wild-type and AIRE-knockdown macrophage-like THP-1 cells stimulated with yeast (MOI 1:2) **(D)** or hyphae (MOI 2:1) **(E)** for 20 or 30 min by Image Stream analysis. Representative images of five independent experiments are shown. **(F)** IL-1β and TNF-α secretion in supernatants from resting and hyphae-stimulated cells. Wild-type (black bar) and AIRE-knockdown (gray bar) macrophage-like THP-1 cells were stimulated with hyphae (MOI 2:1) for 6 h. The horizontal bars denote the mean. Representative graph of three independent experiments. *p < 0.05 as determined by the *t*-test.

Based on the observed delay in FS formation in AIRE-knockdown macrophage-like THP-1 cells, we determined if the phagocytosis of *C. albicans* might be affected. Notably, decreased yeast and hyphal conjugation as well as internalization was observed in AIRE-knockdown macrophage-like THP-1 cells when compared to controls in 20 and 30 min (Figures 5A–C). However, *E. coli* phagocytosis was not affected in AIRE-knockdown macrophage-like THP-1 cells (Figure S5 in Supplementary Material). To determine whether this effect of AIRE was upstream of Syk as previously proposed, yeast and hyphal phagocytosis were measured in AIRE-knockdown macrophage-like THP-1 cells treated with piceatannol, a Syk inhibitor. In this setting, the reduced levels were not further altered by Syk inhibition in AIRE-knockdown macrophage-like THP-1 cells, only in control cells (Figures S6A,B in Supplementary Material), showing that AIRE is important for yeast and hyphal phagocytosis and that it requires a Syk-dependent pathway in macrophage-like THP-1 cells.

Since Dectin-1 is also recruited to the FS after 30 min of hyphal stimulation (Figure 1F) and it is important for lysosomal maturation (33), we determined if lysosomal production was affected by absence of AIRE. Wild-type and AIRE-knockdown macrophage-like THP-1 cells were stimulated with yeast or hyphae for 20 or 30 min. No difference was observed between the wild-type and AIRE-knockdown macrophage-like THP-1 cells stimulated with hyphae. However, simulation of macrophages with yeast resulted in decreased lysosome production in the AIRE-knockdown macrophage-like THP-1 cells at 30 min compared with that in the wild-type macrophage-like THP-1 cells (Figures 5D,E). This suggests that AIRE can be involved in lysosomal production by Dectin-1 in macrophages stimulated with yeast.

Cytokines are pivotal to both innate and adaptive immunity, and their production increases following Dectin ligation. We evaluated IL-1β and TNF-α secretion in wild-type and AIRE-knockdown macrophage-like THP-1 cells stimulated with hyphae. As might be predicted from the interrupted Dectin signaling complex in the absence of normal AIRE function, AIRE-knockdown macrophage-like THP-1 cells secreted lower levels of IL-1β and TNF-α after stimulation with hyphae for 6 h (Figure 5F). This demonstrated that the physiologic inflammatory response after hyphal stimulation requires AIRE in macrophage-like THP-1 cells.

### APECED Patients Macrophages Responses to Fungal Stimulation

**Figure 6:**
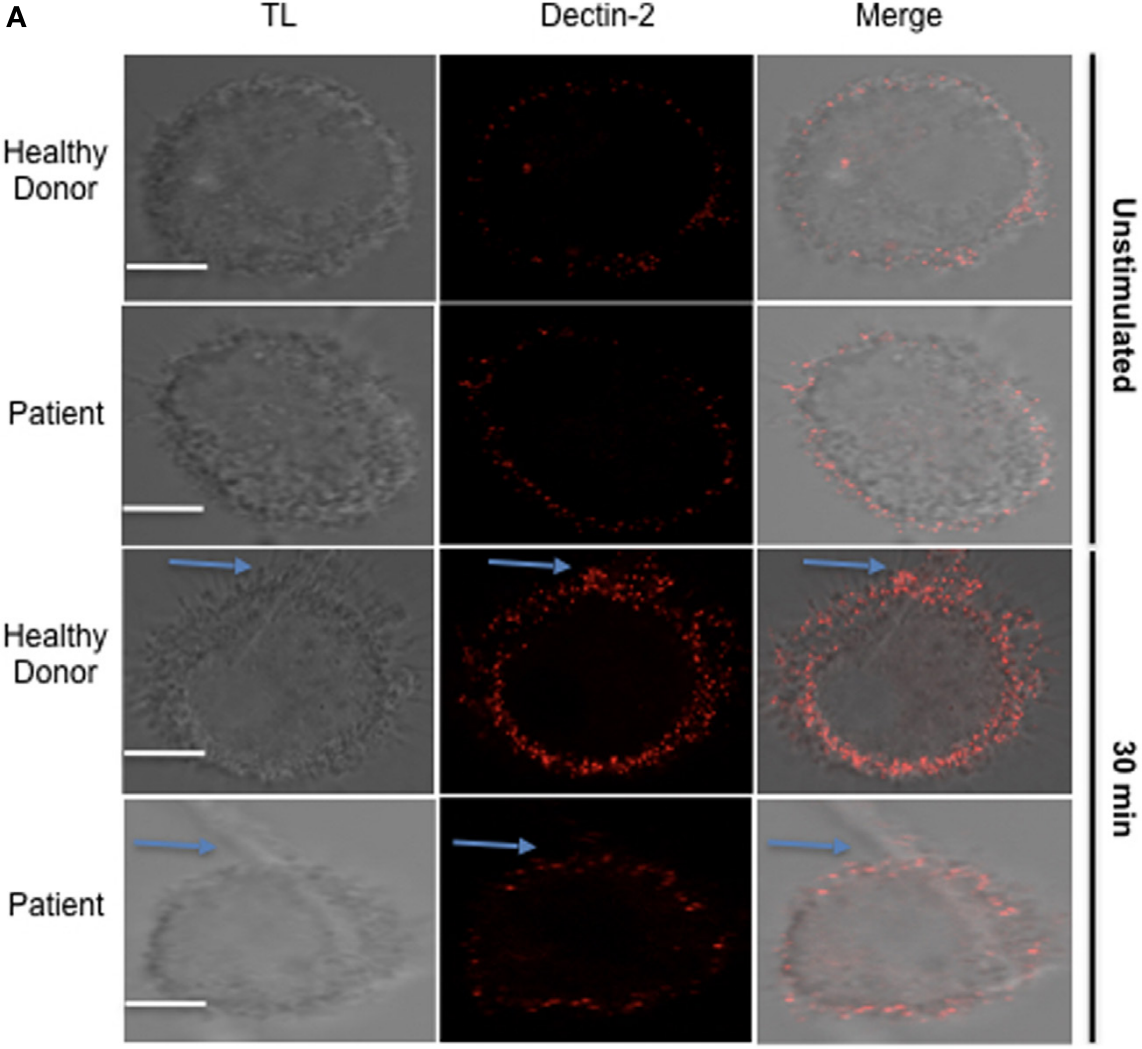
Dectin-2 recruitment in macrophages from healthy donors and autoimmune-polyendocrinopathy-candidiasis-ectodermal dystrophy (APECED) patients after hyphal stimulation. **(A)** Representative confocal micrographs of macrophages from healthy donors and AIRE-deficient patients stimulated with *Candidaalbicans* hyphae for 30 min to observe the recruitment of Dectin-2 (red) receptors to the fungal synapse (FS). The blue arrow indicates the FS. Representative image of 4 independent experiments for both healthy donors and APECED patients, 10 pictures acquired per sample. Scale bar = 5 µm.

**Figure 7:**
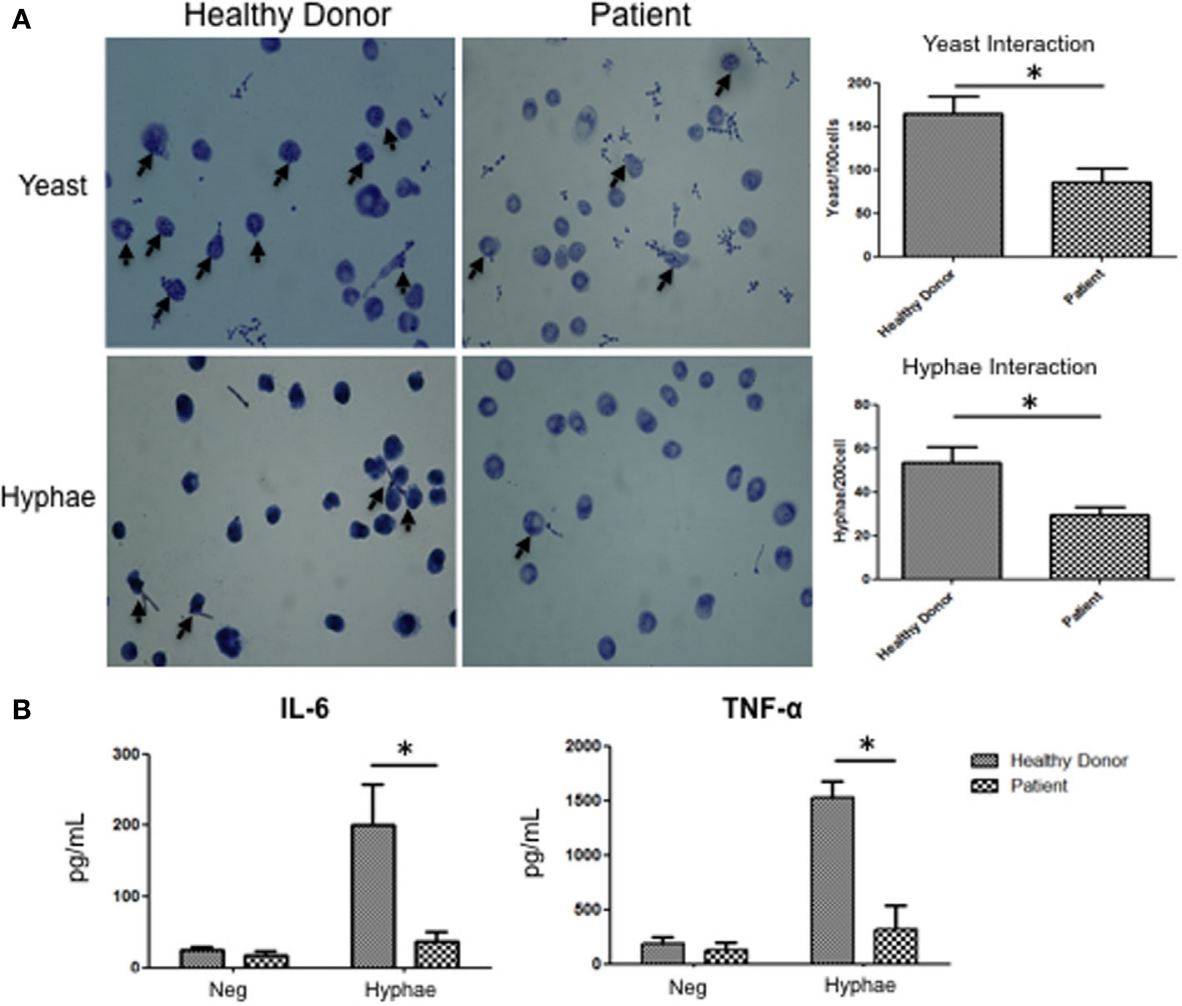
Fungal response in macrophages from autoimmune-polyendocrinopathy-candidiasis-ectodermal dystrophy (APECED) patients. **(A)** Representative images of *C andida albicans* phagocytosis in macrophages from healthy donors and AIRE-deficient patients after 30 min of stimulation with yeast or hyphae. The arrow indicates fungal phagocytosis by macrophages. The images on the right show the numbers of yeast and hyphae inside of the macrophages. **(B)** IL-6 and TNF-α secretion by macrophages from a healthy donor or AIRE-deficient patient in cells unstimulated or stimulated with hyphae for 24 h. **(A,B)** The horizontal bars denote the mean. *p < 0.05 as determined by the *t*-test. Representative image of four independent experiments for both healthy donors and APECED patients.

To determine if those mechanisms are relevant to APECED patients, we evaluated human monocyte-derived macrophages from APECED patients with AIRE mutations as well as from healthy donors. Decreased Dectin-2 expression throughout the entire cell surface of APECED patient macrophages was detected after 30 min of stimulation with *C. albicans* hyphae compared to those from healthy donors (Figure 6). In addition, the APECED macrophages exhibited low phagocytic activity following stimulation with yeast or hyphae for 30 min compared to that in the healthy donor cells (Figure 7A). Macrophages from APECED patients also showed reduced IL-6 and TNF-α secretion after 24 h of hyphal stimulation compared with those from healthy donors (Figure 7B). Importantly, they were not globally impaired as there was no detectable difference in cytokine secretion from those macrophages after LPS stimulation (Figure S7 in Supplementary Material). Thus, the CMC observed in AIRE-deficient patients may be associated with the delay in FS formation and associated defective fungal-specific macrophage signal generation, phagocytosis, and cytokine production.

## Discussion

In this study, we explored the influence of AIRE on Dectin-1 and Dectin-2 dynamic in macrophages stimulated with *C. albicans* hyphae. Although Dectin-1 and Dectin-2 are known to be the key receptors that control the anti-fungal responses against yeast and hyphal forms, respectively (27), the molecular mechanisms underlying the FS formation and function remain unclear. The most important *C. albicans* virulence trait is the reversible capacity to transform into yeast or hyphae. Hyphae promotes invasion, whereas yeast promotes dissemination. They are predominantly recognized by Dectin-2 and Dectin-1, respectively (27, 30). Thus, the process of pathogen recognition may be central to the FS formation, and any ability that it has to modulate the intensity and appropriateness of the response necessary for *C. albicans* destruction. Concomitantly, patients with defects in this recognition process or in Dectin activity are more susceptible to fungal infection (34–36).

Our study shows that FS formation in hyphae-stimulated THP-1 cells is promoted by Dectin-1 and Dectin-2, followed by AIRE, Syk, and CARD9 recruitment when stimulated by *C. albicans* hyphae. These results suggest that a relationship exists between Dectin-1 and Dectin-2 activation and AIRE in macrophages that are activated by hyphae. These receptors are responsible for activation of cytokine secretion, lysosome production, and phagocytic activity in macrophages (3, 27, 33). Given these findings, the absence of AIRE likely results in altered FS formation due to disrupted interactions among AIRE, Dectin-1, Dectin-2, Syk, and CARD9. The FS is important for the recognition of hyphae by macrophages and for the subsequent generation of an effective immune response (3). Because of abnormal FS formation, AIRE-knockdown macrophage-like THP-1 cells exhibited suppressed phagocytosis of yeast and hyphae. This was due to a reduction in activation of the Syk-dependent pathway following Dectin-1 and Dectin-2 ligation, as illustrated in piceatannol-treated THP-1 cells.

The Dectin-1 Syk-dependent pathway is involved in additional immunological functions necessary for the elimination of Candida, such as lysosomal activation. Efficient phagosome maturation is central for the control of candidiasis and is pivotal to both innate and adaptive immunity (33). As we observed, lysosome production in the AIRE-knockdown macrophage-like THP-1 cells stimulated with yeast was reduced; however, this decrease was not observed in the macrophage-like THP-1 cells stimulated with hyphae. These results may be due to the binding of β-glucan to Dectin-1 and the fact that the β-glucan concentration is high in the yeast cell wall and low in the hyphal cell wall. This carbohydrate activates Dectin-1, thereby stimulating lysosome production by the Syk-dependent pathway (3). Moreover, the hyphal form inhibits lysosomal maturation *via* O-mannan, which is present at a high concentration in the cell wall (29). Therefore, reduced lysosome production was only observed in the AIRE-knockdown macrophage-like THP-1 cells stimulated with yeast in this study.

Chronic mucocutaneous candidiasis is frequent in acquired or inherited disorders involving profound T cell defects, especially in those affecting TH17 responses (26, 37). Patients with deficiency in IL-17RA, IL-17F, or ACT1 have defective TH17 function and CMC (11, 14, 18, 26, 37). However, patients with CARD9 deficiency or Dectin-1 Y238X polymorphism are also susceptible to CMC and other fungal infections (8, 15, 35, 38, 39). Defective Dectin-1 expression caused by the Tyr238X polymorphism does not result in immune deficiencies but it is associated with high risk of fungal infections and CMC. This polymorphism generates a truncated Dectin-1 that results in low secretion of IL-1β, IL-6, and TNF-α by peripheral blood mononuclear cell (PBMC), monocytes, and macrophages with impaired IL-17 production in response to *C. albicans* or β-glucan, but normal killing of *C. albicans* by neutrophils (15, 16). On the other hand, CARD9-deficient patients are predisposed to recurrent mucocutaneous and invasive fungal infections with *C. albicans*. These patients show a strong impairment of TNF-α or IL-6 production by neutrophil and monocyte-derived dendritic cells in response to *C. albicans*, whereas IL-17 T-cell production is normal (9, 10, 35).

Autoimmune regulator is essential for proper T cell development and selection in the thymus. While Dectin-1 and CARD9 are expressed in many cell types, studies have shown that AIRE is expressed in peripheral lymphoid tissues, monocytes, and dendritic cells and that it participates in extrathymic functions (23, 24, 40). APECED patients produce variable titers of autoantibodies against IL-17A, IL-17F, or IL-22 associated with CMC (17, 18, 26). However, some patients are susceptible to CMC without these autoantibodies (18), suggesting that other factors may involved in CMC in these patients. AIRE deficiency has been associated with failure in building a proper immune response by monocytes (23, 24, 41). Recently, it has been shown that monocytes from APECED patients have a decrease in IFN-γR2 and STAT1 protein levels that are associated with lower levels of phosphorylated STAT1 molecules after IFN-γ stimulation (24). Another study reported failure of the immune response to *C. albicans*, including a dysregulation of IL-23p19 production in monocytes from APECED patients stimulated with *C. albicans* (41). In 2012, Pedroza et al. established that cytoplasmic AIRE could regulate a Syk-dependent Dectin-1 pathway and secretion of TNF-α in monocytes from APECED patients stimulated with curdlan, a Dectin-1 agonist (23). These results suggest that other Syk-dependent receptors may be affected by AIRE deficiency. Corroborating these findings, here we demonstrate that AIRE-deficient macrophages exhibited less signaling pathway activation at the FS, lower *C. albicans* phagocytosis, and less lysosome formation. On the other hand, CARD9 signaling induces IL-1β, IL-6, and TNF-α secretion by macrophages upon Dectin-1 receptor activation (42). In our study, AIRE-deficient macrophages show CARD9 and Dectin-1, as well as Dectin-2, recruited to the FS followed by decreased secretion of IL-1β, IL-6, and TNF-α. These cytokines are responsible for increasing the intensity of the innate immune response and are involved in generating the TH17 response (7, 43). IL-1β and IL-6 are pivotal to an efficient TH17 response against *C. albicans* (44). TNFα in combination with IL-22 induce innate immune mechanisms in human keratinocytes and maintain the epidermal barrier integrity during *C. albicans* infection (45). Thus, AIRE downstream of Dectin-1 and Dectin-2 engages a critical pathway contributing to antifungal immunity.

In summary, our results show that AIRE is required for the interaction among Dectin-1 and Dectin-2 receptors and Syk-dependent pathway components in human macrophages upon *C. albicans* stimulation. The susceptibility to CMC observed in APECED patients likely includes direct roles of AIRE in peripheral immunity *via* the FS formation and function, which becomes a part of the overall host defense defect. Although APECED is associated with defects in IL-17 immunity caused by autoantibodies, the additional mechanisms we have identified provide additional insight into CMC so frequently observed in these patients.

## Ethics Statement

Blood samples were collected from the patients and healthy donors and were then processed and shipped according to the protocols approved by the Institutional Ethics Committee, the Ministry of Health of Brazil, and the Helsinki Convention.

## Author Contributions

JA and PB designed and conducted the experiments and wrote the manuscript. RM, AC, NZ, LY, CA, and MB-C conducted experiments. JC-D, FW, MD-d-S, and ML-C provided human samples. LP provided technical support. NC and EM designed the experiments. JO and AC-N designed the experiments and wrote and reviewed the manuscript.

## Conflict of Interest Statement

The authors declare that the research was conducted in the absence of any commercial or financial relationships that could be construed as a potential conflict of interest. The reviewer DL and handling editor declared their shared affiliation.

## Supplementary Material

Figure S1 **(A)** Isotype control staining of macrophages THP-1 cells. Representative macrophage-like THP-1 cells stained with AIRE (red), Dectin-1 (purple), Dectin-2 (green), Syk (blue), and its respective IgG control as a negative control for nonspecific staining. **(B)** AIRE overexpression in HEK293T cells. Representative HEK293T cells stained with AIRE (red), nuclei (DAPI, Purple) anti-GFP (green), and respective IgG controls.

Figure S2 Localization of Dectin-1 and Dectin-2 at the FS is independent of the other Dectin receptor activation. **(A)** Representative confocal micrographs of macrophage-like THP-1 cells blocked with anti-Dectin-1, anti-Dectin-2, or IgG control before stimulation with *C. albicans* hyphae for 30 min to observe the recruitment of Dectin-1 (green) or Dectin-2 (red) receptors to the FS. **(B)** Representative confocal micrographs of macrophage-like THP-1 cells blocked with anti-Dectin-1 and anti-Dectin-2 or IgG control before stimulation with *C. albicans* hyphae for 30 min to observe the recruitment of Dectin-1 (green) or Dectin-2 (red) receptors to the FS. **(C,D)** Each dot represents a cell used for the measurement indicated. The horizontal bars denote the mean. **p* < 0.05 as determined by the *t*-test. (C) Relative percentage of total Dectin-1 and Dectin-2 at the FS in macrophage-like THP-1 cells blocked with anti-Dectin-1, anti-Dectin-2 or IgG control. **(D)** Relative percentage of total Dectin-1 and Dectin-2 at the FS in macrophage-like THP-1 cells blocked with anti-Dectin-1 and anti-Dectin-2 or IgG control. Representative confocal micrographs of 30 cells counted in 3 independent experiments.

Figure S3 **(A)** Real-time PCR measurement of *Dectin*-1, *Dectin*-2, *AIRE, Syk*, and *Card9* expression in macrophage-like THP-1 cells stimulated with hyphae for 10, 20, or 30 min. Representative graph of three independent experiments. The horizontal bars denote the mean. **p* < 0.05 as determined by the ANOVA.

Figure S4 Expression of Dectin receptors and proteins from Syk-dependent pathway in wild-type and AIRE-knockdown macrophage-like THP-1 cells. **(A)** Evaluation of AIRE expression in AIRE-knockdown macrophage-like THP-1 cells: wild-type (black line), AIRE-knockdown (dashed line) and IgG control cells (gray line). **(B)** Relative expression of AIRE mRNA (**Left**) and protein expression (**Right**) in AIRE-knockdown and wild-type macrophage-like THP-1 cells. **(C,D)** Expression of Dectin receptors in wild-type (black line) and AIRE-knockdown (dashed line) macrophage-like THP-1 cells and IgG control cells (gray line). **(E)** Syk-dependent pathway activation in macrophage-like THP-1 cells for Dectin-1, Syk, CARD9, and β-actin. Representative image of three independent experiments.

Figure S5 Phagocytic activity in wild-type and AIRE-knockdown macrophage-like THP-1 cells. Wild-type and AIRE-knockdown macrophage-like THP-1 cells were stimulated with *E. coli* (MOI 1:5) for 20 or 30 min. Cells were incubated for 45 min with gentamicin, washed, lysed, and cultured in LB agar. CFU was counted after overnight incubation at 37°C. Representative graph of three independent experiments.

Figure S6 Decreased phagocytic activity resulting from chemical Syk inhibition in THP-1 cells. Wild-type and AIRE-knockdown macrophage-like THP-1 cells were treated with piceatannol for 45 min, followed by stimulation for 30 min with yeast (MOI 1:2) **(A)** or hyphae (MOI 2:1) **(B)** from GFP-positive *C. albicans*. Phagocytosis was assessed with Image Stream. Each dot represents one independent experiment used for the measurement indicated. The horizontal bars denote the mean. **p* < 0.05 as determined by the *t*-test.

Figure S7 Cytokine secretion and *E. coli* phagocytosis in healthy donor or APECED patient macrophages stimulated with LPS. **(A)** Healthy donor or AIRE-deficient patient macrophages stimulated with *E. coli* for 30 min, lysed and cultured in LB agar. CFU was counted after overnight incubation at 37°C. **(B)** IL-6 and TNF-α secretion by healthy donor or AIRE-deficient patient macrophages unstimulated or stimulated with LPS for 24 h. The horizontal bars denote the mean of four independent experiments for both healthy donors or APECED patients.
