# Supplementary figures and images for: The Role of AIRE in the Immunity Against Candida Albicans in a Model of Human Macrophages
Source: Front Immunol. 2018 Mar 21;9:567. doi: 10.3389/fimmu.2018.00567 (PMC5875531; doi:10.3389/fimmu.2018.00567)

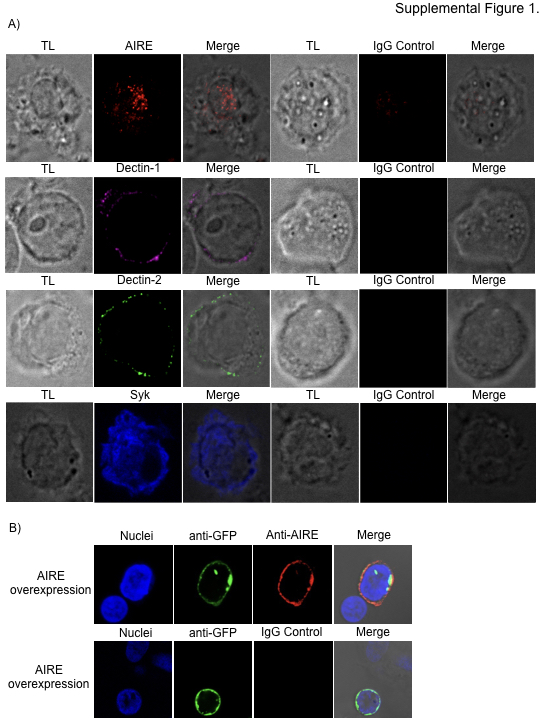

Supplement: Figure S1 — (A) Isotype control staining of macrophages THP-1 cells. Representative macrophage-like THP-1 cells stained with AIRE (red), Dectin-1 (purple), Dectin-2 (green), Syk (blue), and its respective IgG control as a negative control for nonspecific staining. (B) AIRE overexpression in HEK293T cells. Representative HEK293T cells stained with AIRE (red), nuclei (DAPI, Purple) anti-GFP (green), and respective IgG controls. [file image_1.jpeg]

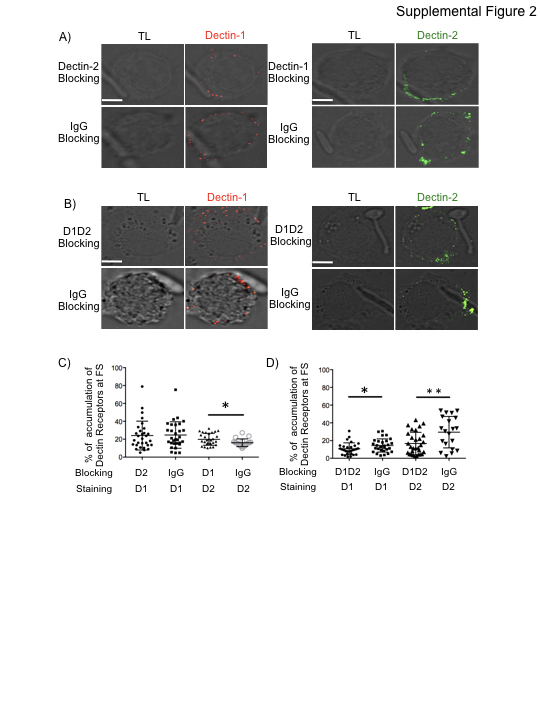

Supplement: Figure S2 — Localization of Dectin-1 and Dectin-2 at the FS is independent of the other Dectin receptor activation. (A) Representative confocal micrographs of macrophage-like THP-1 cells blocked with anti-Dectin-1, anti-Dectin-2, or IgG control before stimulation with C. albicans hyphae for 30 min to observe the recruitment of Dectin-1 (green) or Dectin-2 (red) receptors to the FS. (B) Representative confocal micrographs of macrophage-like THP-1 cells blocked with anti-Dectin-1 and anti-Dectin-2 or IgG control before stimulation with C. albicans hyphae for 30 min to observe the recruitment of Dectin-1 (green) or Dectin-2 (red) receptors to the FS. (C,D) Each dot represents a cell used for the measurement indicated. The horizontal bars denote the mean. *p < 0.05 as determined by the t-test. (C) Relative percentage of total Dectin-1 and Dectin-2 at the FS in macrophage-like THP-1 cells blocked with anti-Dectin-1, anti-Dectin-2 or IgG control. (D) Relative percentage of total Dectin-1 and Dectin-2 at the FS in macrophage-like THP-1 cells blocked with anti-Dectin-1 and anti-Dectin-2 or IgG control. Representative confocal micrographs of 30 cells counted in 3 independent experiments. [file image_2.jpeg]

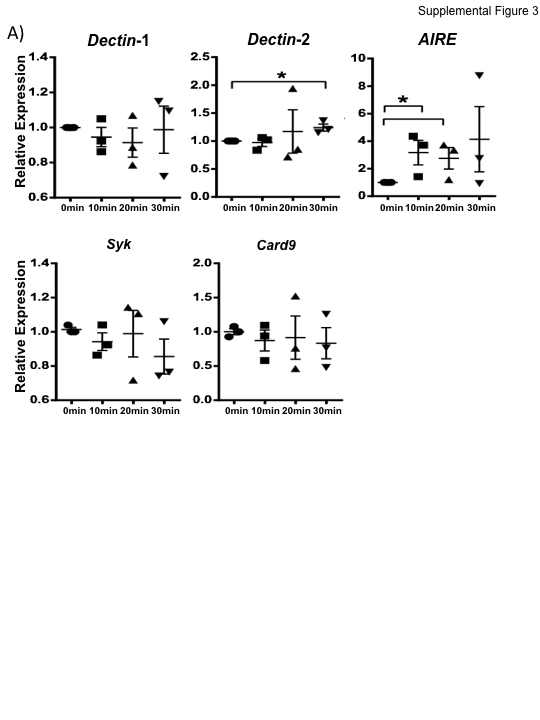

Supplement: Figure S3 — (A) Real-time PCR measurement of Dectin-1, Dectin-2, AIRE, Syk, and Card9 expression in macrophage-like THP-1 cells stimulated with hyphae for 10, 20, or 30 min. Representative graph of three independent experiments. The horizontal bars denote the mean. *p < 0.05 as determined by the ANOVA. [file image_3.jpeg]

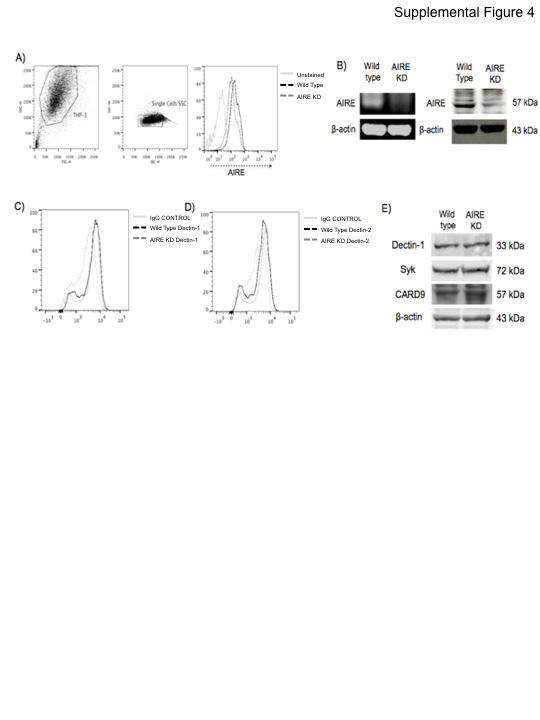

Supplement: Figure S4 — Expression of Dectin receptors and proteins from Syk-dependent pathway in wild-type and AIRE-knockdown macrophage-like THP-1 cells. (A) Evaluation of AIRE expression in AIRE-knockdown macrophage-like THP-1 cells: wild-type (black line), AIRE-knockdown (dashed line) and IgG control cells (gray line). (B) Relative expression of AIRE mRNA (Left) and protein expression (Right) in AIRE-knockdown and wild-type macrophage-like THP-1 cells. (C,D) Expression of Dectin receptors in wild-type (black line) and AIRE-knockdown (dashed line) macrophage-like THP-1 cells and IgG control cells (gray line). (E) Syk-dependent pathway activation in macrophage-like THP-1 cells for Dectin-1, Syk, CARD9, and β-actin. Representative image of three independent experiments. [file image_4.jpeg]

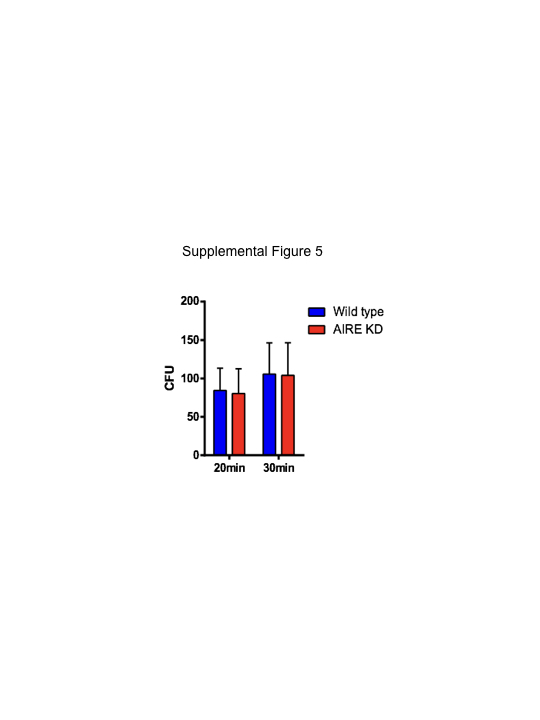

Supplement: Figure S5 — Phagocytic activity in wild-type and AIRE-knockdown macrophage-like THP-1 cells. Wild-type and AIRE-knockdown macrophage-like THP-1 cells were stimulated with E. coli (MOI 1:5) for 20 or 30 min. Cells were incubated for 45 min with gentamicin, washed, lysed, and cultured in LB agar. CFU was counted after overnight incubation at 37°C. Representative graph of three independent experiments. [file image_5.jpeg]

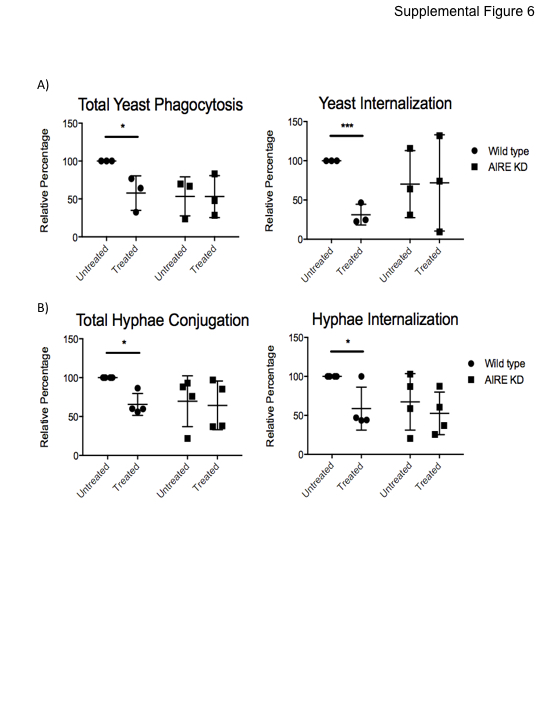

Supplement: Figure S6 — Decreased phagocytic activity resulting from chemical Syk inhibition in THP-1 cells. Wild-type and AIRE-knockdown macrophage-like THP-1 cells were treated with piceatannol for 45 min, followed by stimulation for 30 min with yeast (MOI 1:2) (A) or hyphae (MOI 2:1) (B) from GFP-positive C. albicans. Phagocytosis was assessed with Image Stream. Each dot represents one independent experiment used for the measurement indicated. The horizontal bars denote the mean. *p < 0.05 as determined by the t-test. [file image_6.jpeg]

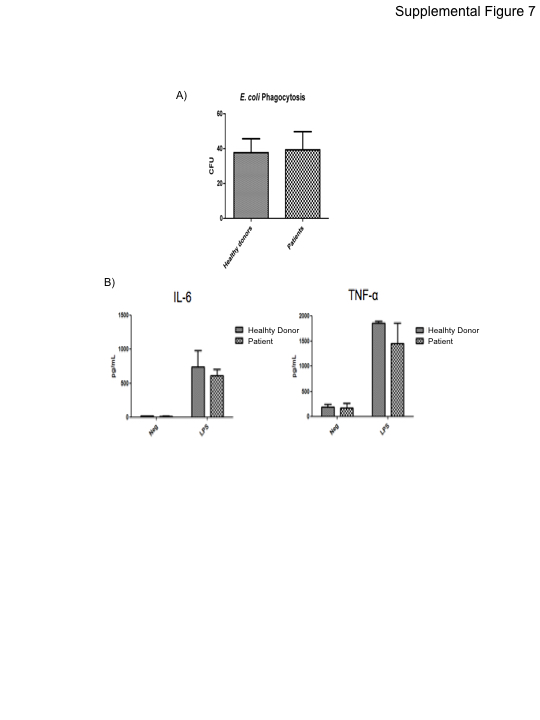

Supplement: Figure S7 — Cytokine secretion and E. coli phagocytosis in healthy donor or APECED patient macrophages stimulated with LPS. (A) Healthy donor or AIRE-deficient patient macrophages stimulated with E. coli for 30 min, lysed and cultured in LB agar. CFU was counted after overnight incubation at 37°C. (B) IL-6 and TNF-α secretion by healthy donor or AIRE-deficient patient macrophages unstimulated or stimulated with LPS for 24 h. The horizontal bars denote the mean of four independent experiments for both healthy donors or APECED patients. [file image_7.jpeg]
